# Trajectory as the Diagnostic Signal: Persistent Multifactorial Anemia Leading to a Diagnosis of Coeliac Disease

**DOI:** 10.1002/ccr3.73915

**Published:** 2026-11-11

**Authors:** Chibuzo C. Manafa, Chukwuzitelu O. Okafor‐Udah, Nnamdi Okoli, Stephen Adilih

**Affiliations:** ^1^ Department of Family Medicine Queensland Medical Clinic Calgary Alberta Canada; ^2^ University of Liverpool Hospitals Liverpool UK

**Keywords:** coeliac disease, iron deficiency, malabsorption, multifactorial anemia, persistent anemia

## Abstract

A man in his late 50s with no documented prior medical history and no laboratory contact for several years presented with inferior ST‐elevation myocardial infarction treated with percutaneous coronary intervention. Initial bloodwork showed hemoglobin of 170–182 g/L with normal red cell indices, possibly reflecting haemoconcentration during acute illness. Hemoglobin then declined to 126–130 g/L and remained persistently low. The decline was accompanied by transient macrocytosis, vitamin B12 deficiency, severe folate deficiency, and progressive iron‐store depletion, with ferritin falling from 186 to 55 μg/L alongside falling serum iron and transferrin saturation, although these values stayed within reference ranges. Repletion of B12 and folate normalized the mean corpuscular volume but produced negligible hemoglobin recovery, and a reticulocyte production index of approximately 0.65 confirmed a hypoproliferative marrow response. Renal function was preserved, serum protein electrophoresis was unremarkable, and haemolysis was not supported by the overall pattern. Total IgA was preserved, anti‐tissue transglutaminase IgA exceeded 2500 KIU/L (upper limit of normal 15.0 KIU/L; > 166× ULN), and duodenal biopsy demonstrated intraepithelial lymphocytosis with villous blunting. Five months after starting a gluten‐free diet, hemoglobin normalized to 142 g/L for the first time in the documented course, anti‐tissue transglutaminase IgA fell to 54.1 KIU/L, and transferrin saturation rose to 0.27. Ferritin remained low at 51 μg/L, consistent with recovering erythropoiesis before iron stores are replete. The trajectory, not any single value, was the diagnostic signal: persistent anemia after B12 and folate correction, with iron stores falling inside the reference range, should prompt coeliac testing.

## Key Clinical Message

Persistent anemia despite correction of vitamin B12 and folate deficiency, particularly with progressively declining iron stores, should prompt investigation for coeliac disease. This applies even when gastrointestinal symptoms are absent and iron indices remain within laboratory reference ranges.

## 1 Introduction

Coeliac disease (CD) affects approximately 1%–2% of the general population and remains underdiagnosed in adults, in whom extraintestinal presentations are common and anemia is one of the most frequent clinical clues [1, 2]. CD is an immune‐mediated enteropathy triggered by dietary gluten in genetically susceptible individuals, nearly all of whom carry HLA‐DQ2 or HLA‐DQ8. Gluten peptides deamidated by tissue transglutaminase 2 drive a mucosal T cell response that produces villous atrophy and malabsorption, and the same enzyme is the autoantigen targeted by the antibodies used in diagnosis [1]. Anemia in CD may be multifactorial, reflecting iron malabsorption, concurrent vitamin B12 and folate deficiency, and inflammatory effects on erythropoiesis and iron handling [2, 3, 4, 5]. For this reason, correction of a single documented deficiency may not produce full hematological recovery when the underlying process is malabsorptive.

Previously silent CD has been described as becoming apparent after physiological stressors, most often abdominal surgery, with scattered reports after viral infection [6, 7, 8, 9]. In the present case, the cardiac admission provided the first laboratory baseline in several years, so whether the infarction triggered the disease or simply preceded its detection cannot be determined.

We report a case in which follow‐up after acute coronary syndrome requiring percutaneous coronary intervention (PCI) revealed a hematological trajectory of persistent anemia, corrected B12 and folate deficiency without hemoglobin recovery, and progressive iron‐store depletion, ultimately leading to diagnosis of biopsy‐confirmed CD with subsequent hematological and serological response to a gluten‐free diet. This case shows the diagnostic value of serial laboratory trends when individual results remain non‐specific or within conventional reference ranges.

## 2 Case History and Examination

A man in his late 50s with no documented prior medical history and no laboratory testing on electronic records for several years presented to the Emergency Department in December 2024 with inferior ST‐elevation myocardial infarction. He underwent urgent PCI with drug‐eluting stent implantation to the left posterior descending artery and posterolateral branch. Initial laboratory testing during this admission therefore provided the first available hematological reference point in several years.

He had no personal or family history of gastrointestinal or autoimmune disease and denied diarrhea, steatorrhoea, haematochezia, weight loss, or other alarm gastrointestinal symptoms throughout follow‐up. After discharge, he was treated with dual antiplatelet therapy and guideline‐directed secondary prevention, including ticagrelor, aspirin, beta‐blocker therapy, angiotensin‐converting enzyme inhibitor therapy, high‐intensity statin therapy, and ezetimibe. He was not prescribed a proton pump inhibitor or metformin at any point in the disease course. He had no history of hypertension, diabetes mellitus, dyslipidaemia, or tobacco use, and no family history of premature coronary artery disease. He had no laboratory contact for several years before the index admission, so no pretreatment lipid profile was available. Cardiology follow‐up documented elevated lipoprotein(a) of 156 nmol/L, supporting an identified atherosclerotic risk context for the obstructive coronary event.

## 3 Methods (Investigations and Differential Diagnosis)

Initial bloodwork during the index cardiac admission showed hemoglobin of 170–182 g/L, hematocrit of 0.51–0.55, normal MCV of 93–94 fL, and platelets of 234–263 × 10^9^/L, measured during acute cardiac illness and therefore possibly affected by haemoconcentration. By March 2025, hemoglobin had fallen to 129 g/L, and it remained persistently low at 126–130 g/L over the following year. Platelets rose to 363 × 10^9^/L in March 2025, remaining within the reference range, before gradually returning toward baseline; reactive thrombocytosis is recognized as an indirect marker of iron deficiency [10].

At six to seven months, MCV rose transiently to 103 fL, coinciding with vitamin B12 deficiency of 152 pmol/L and severe folate deficiency below 1.5 nmol/L. After repletion, vitamin B12 increased to 307 pmol/L and folate to greater than 45.4 nmol/L in October 2025, with normalization of MCV to 92–93 fL. Hemoglobin recovery, however, was negligible, remaining 126–130 g/L despite biochemical correction of both deficiencies.

Serial iron studies showed progressive iron‐store depletion despite values remaining within or at the lower end of the laboratory reference range. Ferritin declined from 186 μg/L in June 2025 to 74 μg/L in November 2025, and later to 55 μg/L in April 2026. Over the same period, serum iron declined from 20 to 11 μmol/L, and transferrin saturation fell from 0.37 to 0.22. Repeated fecal immunochemical tests were negative. Renal function was preserved, with eGFR consistently above 90 mL/min/1.73 m^2^ and repeatedly normal urine albumin‐to‐creatinine ratios. Serum protein electrophoresis was unremarkable. Blood smear showed unremarkable RBC morphology. Polyspecific direct antiglobulin testing was negative. LDH was mildly elevated at 347 U/L, but haptoglobin was not reduced and the reticulocyte response was hypoproliferative, with a reticulocyte production index of approximately 0.65, well below the value of 2.0 generally taken to indicate an adequate marrow response [11]. hsCRP was 1.0 mg/L in March 2025 and 0.3 mg/L in April 2026, and overall, haemolysis and a dominant systemic inflammatory state were not supported by the pattern of results.

Medication‐associated vitamin B12 deficiency was considered unlikely, as the patient was not prescribed metformin or a proton pump inhibitor at any point in the disease course.

Given persistent anemia after B12 and folate correction, progressive iron‐store depletion, and absence of an identified bleeding source, coeliac serology was obtained on 5 December 2025. Total IgA, measured in November 2025, was preserved at 5.43 g/L, supporting interpretation of IgA‐based serology. Anti‐tissue transglutaminase IgA exceeded 2500 KIU/L (upper limit of normal 15.0 KIU/L), more than 166 times the upper limit of normal, a titre well above the ≥ 10× upper limit of normal threshold that, although derived in pediatric guidance, is associated with high positive predictive value for Marsh Grade 3 histological change [12], prompting referral for upper gastrointestinal endoscopy and gastroenterology review. Direct‐to‐procedure thin‐scope upper endoscopy was performed on 17 March 2026 and showed duodenal oedema, erythema and scalloping. Protocol biopsies comprised four specimens from the second portion of the duodenum and two from the duodenal bulb. Histology demonstrated intraepithelial lymphocytosis and villous blunting at both sites, with active inflammation additionally noted in the second portion, confirming coeliac enteropathy. The full longitudinal laboratory trajectory is summarized in Table 1, and the clinical course with the corresponding hemoglobin trajectory is shown in Figure 1.

**TABLE 1:** Longitudinal laboratory trajectory from the index cardiac admission through serology, endoscopic diagnosis, and posttreatment assessment five months after initiation of a gluten‐free diet.

Parameter	Dec 2024	Mar 2025	Jun–Jul 2025	27 Oct 2025	19 Nov 2025	5 Dec 2025	Apr 2026	Sep 2026
Hemoglobin, g/L	170–182	129	127	—	127	130	128	142
Hematocrit	0.51–0.55	0.38	0.39	—	0.40	0.39	0.39	0.44
MCV, fL	93–94	90	103	—	92	93	89	89
RDW, %	14.1–14.2	14.9	13.9	—	13.2	14.1	14.8	14.7
Platelets, ×10^9^/L	234–263	363	345	—	297	258	307	270
Ferritin, μg/L	—	—	186	—	74	—	55	51
Serum iron, μmol/L	—	20	13	—	—	—	11	15
TIBC, μmol/L	—	—	—	—	—	—	49	55
Transferrin saturation	—	0.37	0.29	—	—	—	0.22	0.27
Vitamin B12, pmol/L	—	231	152	307	—	—	306	328
Folate, nmol/L	—	—	< 1.5	> 45.4	—	—	—	11.0
Reticulocytes/RPI	—	—	—	—	1.2%/~0.65	—	—	—
hsCRP, mg/L	—	1.0	—	—	—	—	0.3	—
Total IgA, g/L	—	—	—	—	5.43	—	—	—
Anti‐tTG IgA, KIU/L	—	—	—	—	—	> 2500	—	54.1

*Note:* Anti‐tTG IgA has an upper limit of normal of 15.0 KIU/L. Column dates reflect the true collection date of each value. April 2026 values were obtained before the gluten‐free diet was started. September 2026 values were obtained approximately five months after initiation of a gluten‐free diet with concurrent iron and vitamin repletion. Oral iron intake throughout the pretreatment observation period was self‐reported, intermittent and could not be quantified.

Abbreviations: anti‐tTG IgA, anti‐tissue transglutaminase immunoglobulin A; hsCRP, high‐sensitivity C‐reactive protein; IgA, immunoglobulin A; MCV, mean corpuscular volume; RDW, red cell distribution width; RPI, reticulocyte production index; TIBC, total iron binding capacity.

**FIGURE 1:**
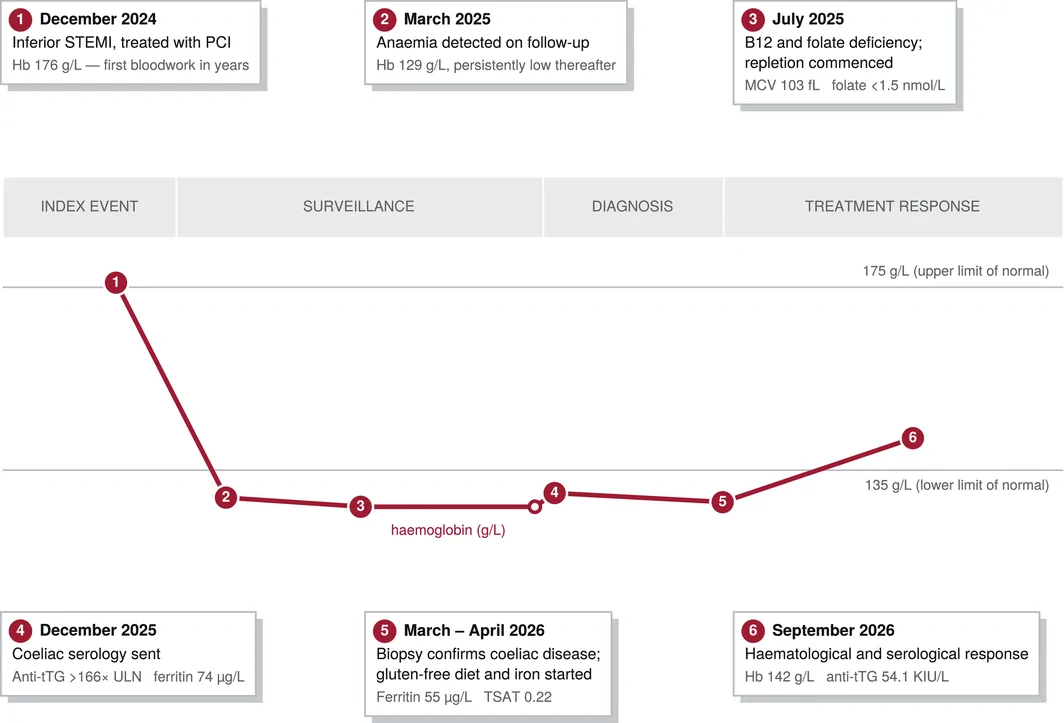
Clinical course and hemoglobin trajectory. Numbered points on the hemoglobin trace correspond to the numbered panels. Horizontal rules mark the laboratory reference limits. The December 2024 value is plotted at the midpoint of the recorded 170–182 g/L range. The unlabelled point is the November 2025 measurement. Full laboratory data are given in Table 1. Anti‐tTG, anti‐tissue transglutaminase IgA; Hb, hemoglobin; MCV, mean corpuscular volume; TSAT, transferrin saturation; ULN, upper limit of normal.

## 4 Conclusion and Results (Treatment, Outcome, and Follow‐Up)

Initial management focused on correction of the documented nutritional deficiencies while the anemia pattern was being followed. Intramuscular cyanocobalamin 1000 μg and oral folic acid 5 mg daily were commenced in July 2025 after vitamin B12 and severe folate deficiency were identified. Oral iron supplementation was also recommended, but intake was self‐reported, intermittent, and could not be quantified.

After coeliac serology in December 2025 returned strongly positive, the patient was referred for upper gastrointestinal endoscopy and gastroenterology review. Duodenal biopsies in March 2026 confirmed coeliac enteropathy. The patient was reviewed by gastroenterology in April 2026 and repeat bloods were taken before treatment began, after which the coeliac treatment plan was initiated, comprising commencement of a strict gluten‐free diet under coeliac‐trained dietitian supervision, continuation of cyanocobalamin and folic acid supplementation, full oral iron repletion, multivitamin, and calcium and vitamin D supplementation, vaccination review, and first‐degree relative screening, in line with current guidance. Those bloods showed hemoglobin of 128 g/L with MCV of 89 fL, ferritin of 55 μg/L, transferrin saturation of 0.22, serum iron of 11 μmol/L, and vitamin B12 of 306 pmol/L.

Reassessment about five months after the gluten‐free diet was started, with concurrent iron and vitamin repletion, showed hemoglobin of 142 g/L. This was the first value within the laboratory reference range since the index admission. Hematocrit was 0.44, MCV 89 fL, serum iron 15 μmol/L, total iron binding capacity 55 μmol/L and transferrin saturation 0.27. Anti‐tissue transglutaminase IgA fell from > 2500 KIU/L to 54.1 KIU/L. Ferritin remained low at 51 μg/L, and iron repletion continues. Vitamin B12 was 328 pmol/L and folate 11.0 nmol/L on continued supplementation. The patient remains under active follow‐up, with repeat serology and iron indices scheduled and repeat upper endoscopy at two to three years discussed to assess mucosal healing.

## 5 Discussion

The diagnosis here emerged from a pattern rather than from any single result. The patient had no diarrhea, weight loss, or overt gastrointestinal bleeding, and no early laboratory value pointed to coeliac disease on its own. What pointed to it was serial discordance: anemia that persisted after B12 and folate were corrected, iron stores that fell steadily while remaining within reference ranges, and a hypoproliferative reticulocyte response.

The differential was worked through in sequence. Occult gastrointestinal blood loss on dual antiplatelet therapy was the first consideration and was argued against by repeatedly negative fecal immunochemical testing, by a gradual decline that was not confined to the peri‐procedural period, and by the absence of a bleeding source at upper endoscopy. Anemia of inflammation was unlikely at an hsCRP of 1.0 and 0.3 mg/L, renal anemia was excluded by an eGFR consistently above 90 mL/min/1.73 m^2^, haemolysis by a preserved haptoglobin and a negative direct antiglobulin test, and a plasma cell dyscrasia by unremarkable protein electrophoresis. Isolated nutritional deficiency remained until correction of B12 and folate failed to restore the hemoglobin, at which point a malabsorptive process became the unifying explanation.

The phenomenon of CD becoming apparent following a physiological stressor, often described as unmasking or triggering, is well described [6, 7, 8]. Most reports have followed abdominal surgery, with scattered descriptions after viral infection [9]. The closest cardiac scenario we identified was a 2023 report of CD diagnosed after myocardial infarction with non‐obstructive coronary arteries, in which CD was proposed as a possible prothrombotic contributor to the infarction itself [13]. Our case differs in clinical framing. The patient had obstructive atherosclerotic coronary disease requiring PCI, with elevated lipoprotein(a) providing an identified cardiovascular risk context. CD was not advanced as the cause of the infarction, and no causal relationship between the cardiac event and the enteropathy is proposed.

The hematological trajectory was informative for three reasons. First, the hemoglobin fall was anchored to repeated high‐normal values during the index admission. A hemoglobin of 126–130 g/L in isolation might have been interpreted as mild, non‐specific anemia after acute illness. In this case, the fall from the index values created a visible direction of travel. Second, the transient rise in MCV to 103 fL coincided with B12 deficiency and severe folate deficiency, but normalization of MCV after repletion did not produce meaningful hemoglobin recovery. This fits the known complexity of CD‐associated anemia, in which iron malabsorption may coexist with B12 and folate deficiency [2, 3, 4, 5]. Concurrent multi‐deficiency anemia has been documented in adult CD cohorts [14]. Although inflammatory effects on erythropoiesis and iron handling are recognized mechanisms [4, 5], this patient's hsCRP was 1.0 mg/L and 0.3 mg/L on available testing, making a dominant systemic inflammatory or hepcidin‐mediated explanation less likely.

Third, the iron profile was more informative as a trajectory than as a threshold‐crossing result. Ferritin declined from 186 μg/L to 55 μg/L, while serum iron declined from 20 to 11 μmol/L and transferrin saturation from 0.37 to 0.22. These values remained at or near the lower end of laboratory reference ranges throughout; hsCRP was low, and repeated FIT testing was negative. The patient did not have classical iron deficiency anemia. What mattered was that iron stores and iron availability fell steadily alongside persistent hypoproliferative anemia. Observational work has described low ferritin without anemia as a common feature of screen‐detected adult CD [15], and a selected retrospective cohort of 86 individuals with absolute iron deficiency, defined by ferritin below 50 μg/L, found confirmed CD in 14% irrespective of anemia status [16]. The present case approaches rather than meets that definition, with a pretreatment nadir of 55 μg/L, and is consistent with the broader observation that iron‐store depletion in CD may be evident before conventional diagnostic thresholds are crossed.

The posttreatment assessment completes the picture. Anti‐tissue transglutaminase IgA fell by more than an order of magnitude, giving objective evidence of dietary response, and hemoglobin normalized for the first time in the documented course. Hemoglobin and transferrin saturation improved while ferritin remained low, indicating hematological recovery before complete restoration of iron stores. This response to gluten withdrawal supports the view that the preceding decline reflected duodenal malabsorption rather than an unrelated process.

Coeliac serology should be considered in adults with persistent anemia and sequential nutritional deficiencies, even when gastrointestinal symptoms are absent and iron indices have not crossed conventional diagnostic thresholds. Current guidance recommends coeliac testing in adults with unexplained iron, vitamin B12 or folate deficiency [17]. This case supports applying that recommendation once the pattern becomes multi‐nutrient and directionally abnormal. The relevance extends beyond gastroenterology: unexplained anemia is common during post‐ACS follow‐up, and when serial values keep drifting after the acute period, a persistent multi‐nutrient pattern should not be attributed automatically to the cardiac admission, antiplatelet therapy, or other peri‐procedural factors.

Prior studies have noted associations between coeliac disease and cardiovascular disease [18], and we examined the mechanisms proposed for them against this patient's own data. In a Swedish cohort of 28,190 individuals with biopsy‐verified coeliac disease, the hazard ratio for incident ischaemic heart disease was 1.19 (95% CI 1.11 to 1.28), with a comparable excess in those who had inflammation without villous atrophy [19]. Anemia is the most direct of these mechanisms, since it worsens the balance between myocardial oxygen supply and demand, and low hemoglobin after acute coronary syndrome is an independent predictor of major adverse cardiovascular events [20]. This patient's hemoglobin remained at 126–130 g/L from three months after his infarction until treatment began. Malabsorption of folate and vitamin B12 raises homocysteine, which was elevated in 20% of adults at coeliac diagnosis in one prospective series and normalized on a gluten‐free diet [21]. Both deficiencies were documented here, although homocysteine was not measured. Chronic systemic inflammation is the third mechanism usually cited and the least supported in this patient, whose hsCRP was 1.0 and 0.3 mg/L.

This is a single‐case observation and the trajectory described may not generalize. Oral iron intake was self‐reported, intermittent and could not be quantified, and iron repletion since April 2026 has been concurrent with the gluten‐free diet, so the relative contribution of each to the hematological response cannot be separated. The starting hemoglobin of 170–182 g/L was measured during acute cardiac illness and may partly reflect haemoconcentration rather than the patient's true pre‐illness baseline. Posttreatment follow‐up remains short at approximately five months; ferritin has not yet normalized, and longer‐term serology and iron indices will be required to document sustained mucosal and hematological recovery.

## Author Contributions

**Chibuzo C. Manafa:** conceptualization, investigation, writing – original draft, methodology, validation, visualization, writing – review and editing, project administration, data curation, supervision, resources. **Chukwuzitelu O. Okafor‐Udah:** writing – review and editing, visualization, validation, methodology, resources. **Nnamdi Okoli:** investigation, methodology, validation, visualization, writing – review and editing, supervision, resources. **Stephen Adilih:** supervision, resources, visualization, writing – review and editing, validation, methodology, investigation.

## Funding

The authors have nothing to report.

## Ethics Statement

Written informed consent was obtained from the patient for publication of this case report and all accompanying clinical data. The report adheres to CARE guidelines.

## Consent

Written informed consent was obtained from the patient to publish this report in accordance with the journal's patient consent policy.

## Conflicts of Interest

The authors declare no conflicts of interest.

## Data Availability

The data that support the findings of this study are available on request from the corresponding author. The data are not publicly available due to privacy or ethical restrictions.
